# Gender Disparities in Mental Health and Study Demands among Medical Students: A Three-Cohort Study at Two German Universities

**DOI:** 10.5334/pme.1899

**Published:** 2025-12-04

**Authors:** Jan C. Zoellick, Susanne Dettmer, Amanda S. Voss

**Affiliations:** 1 Charité – Universitätsmedizin Berlin, corporate member of Freie Universität Berlin and Humboldt-Universität zu Berlin, Institute of Medical Sociology and Rehabilitation Science, Charitéplatz 1, 10117 Berlin, Germany; 2 German Center for Mental Health (DZPG), partner site Berlin/Potsdam, Germany; 3 Friedrich-Alexander-Universität Erlangen-Nürnberg, Institute and Outpatient Clinic of Occupational, Social, and Environmental Medicine, Henkestraße 9-11, 91054 Erlangen, Germany

## Abstract

**Background:**

Medical students regularly report poorer mental health outcomes than students from other faculties and in other degree programmes. Gender and year of study have been discussed as factors influencing mental health. However, study results for both factors are inconclusive. Moreover, little is known about the associations between gender and study progression with perceived study demands.

**Methods:**

The purpose of this study was to analyse medical students’ self-reported mental health status and perceived study demands with particular focus on gender differences and study progression. We conducted an online survey in two German universities across three data collection waves during the winter terms of the academic years 2021, 2022, and 2023. We included 1,083 medical students (73% women) in our analyses: bivariate *t*-tests and ANOVAs with gender, study progression, and university as predictors of depression, exhaustion, six dimensions of study demands, and satisfaction with learning and study.

**Results:**

Our results demonstrated gender differences regarding mental health and study demands, favouring men on all outcomes in *t*-tests and ANOVAs. We did not find differences between beginners and advanced students in mental health, but beginners reported greater difficulty with performance pressure and self-structuring.

**Conclusions:**

Our findings confirm persistent gender differences in mental health and study demands, with women reporting higher distress. We did not find differences in terms of satisfaction with studies or learning. To improve medical students’ well-being, we suggest targeted interventions for vulnerable groups using gender-sensitive approaches and designs. Beginners likely benefit from support with self-structuring and managing performance pressure.

## Introduction

Medical students regularly report poorer mental health outcomes than students from other faculties and in other degree programmes [1,2]. In attempts to explain these struggles of medical students, scholars stress structural and personal factors. The medical degree structure includes academic and practical parts that are emotionally and organisationally challenging as well as learning-intensive exams [3]. Medicine is a highly distinctive, status-oriented field of study that exhibits a strong work ethic [4,5]. Accordingly, medical students often exhibit strong performance motives and a tendency to overexert themselves [6,7]. Consequently, burnout [8,9] and depression scores [10] are often pronounced and exceed clinical thresholds in this target group. The prevalence of burnout among medical students has been estimated at 37% (95%-CI: 33%–42%) in a meta-analysis of 42 studies and 26,824 students [11] and at 44% (95%-CI: 33%–55%) in a meta-analysis of 24 studies and 17,431 students [8]. For depression, the prevalence was 28% (95%-CI: 24%–32%) in a meta-analysis with 72 studies and 62,728 students [10]. In each case, medical students report a higher prevalence of mental health difficulties than the general public [12]. Impaired mental health may both contribute to and result from increased study demands, leading to reduced academic performance and study satisfaction, interruptions or delays, and, finally, dropouts [13,14]. Thus, it is important to understand student mental health to intervene early, providing individual support for struggling students whilst also addressing structural issues at the university-wide level, e.g., reducing course workloads, providing adequate learning environments, or installing student mental health counselling [14]. Even though medical studies seem to be comparably demanding [1], the specific study demands do not affect all students equally. A range of sociodemographic variables are associated with mental health and perceived study demands; among them are gender, age, migration history, and parental academic background [15,16]. However, in contrast to those well-established influences, the relationships between gender, study progression, mental health, and perceived study demands have not yet been sufficiently explored and understood [10].

### Gender

For the association between gender and mental health, two contrasting frameworks have been proposed. The first approach identifies women and girls as disadvantaged based on prevalent gender roles, expectations, and experiences [17]. Accordingly, women and girls are more likely than boys and men to experience adverse events such as discrimination, bullying, and sexual violence, which can cause emotional distress and trauma that can lead them into therapy [18,19]. Additionally, the hegemonic ideal of femininity relies on emotional sensitivity and submissiveness, which leads women to internalise problems [17]. Academically, particularly in the natural sciences, essentialist views on intelligence and ability prevail, to the detriment of female students who experience higher levels of impostorism and lower confidence in their achievements [20]. These essentialist institutional cultures may hinder women’s performance. In contrast, the hegemonic ideal of masculinity, with traits such as assertiveness and independence, promotes externalisation of problems [17]. This conception of masculinity seems protective of noticing mood disorders. In sum, the first approach expects poorer mental health, more academic struggles, and more help-seeking behaviour in women than in men.

The second approach posits a “crisis of manhood/boyhood” particularly regarding mental health in school and academic performance. This perspective focuses on the downsides of the hegemonic ideal of masculinity, which impairs men’s help-seeking behaviour instead of making them more resilient against mental health difficulties [21]. Consequently, men are less likely to approach professionals for their mental health [22]. Additionally, the suicide mortality rate among men is approximately 2.5 times higher than women’s despite a lower prevalence of suicide attempts [23]. Academically, meta-analyses found small but significant gender effects favouring girls in school and academic performance across fields [24,25]. In Germany, female students outnumber males in highly distinctive and competitive fields such as medicine at least 60:40 since 2005 and psychology at least 70:30 since 1998 [26]. Explanations focus on the misfit between school and academic demands on the one hand and essentialist masculine traits and behaviours on the other hand [27]. More granular research has identified within-gender personality differences as a powerful lens to understand underachievement [28]. In sum, the second approach expects worse mental health and more academic struggles in men with less help-seeking behaviour than in women. Empirical results in meta-analyses for the field of medicine provide inconclusive evidence – for burnout, Almutairi et al. [11] found higher rates in female medical students than males, whereas Frajerman et al. [8] found no gender effects; for depression, Puthran et al. [10] did not find effects for gender. Thus, further studies are needed to assess gender disparities.

### Study progression

As they progress through their studies, students encounter various stressors. In year one, students develop new social contexts, discover the mode and language of science, need to self-structure and organise their learning, and cope with high workloads [29]. Students typically either habituate to these stressors and develop problem-focused coping strategies [30] or discontinue their studies. Accordingly, struggles following study demands should decrease with study progression because of personal development and selection bias in higher years of study. As their studies progress, medical students are increasingly involved in medical practice, encountering emotionally challenging situations as new stressors. Research on the Job Demand Control Model [31] demonstrates that clinical practice is characterised by high demands coupled with a loss of control, leading to high burnout rates and emotional struggles [32]. Accordingly, mental health is expected to decrease as the study progresses. This trend, however, can be counteracted by personal growth and development driven by experience and ageing, which covary with study progression [11]. Empirical results are inconsistent. Almutairi et al. [11] reported a declining trend in burnout with study progress, however with limited confidence, as study progress covaries with age; Frajerman et al. [8] did not report results on study progress or burnout. For depression, Puthran et al. [10] found a significant decline in depression scores between year 1 (31%) and year 5 (21%) of medical studies. In contrast, Rotenstein et al. [33] found a 13% increase from the beginning to the end of medical school across 9 longitudinal studies. A recent longitudinal study found alleviated depression scores, but worsened burnout scores in study progression between years 1, 3 and 6 [29].

### Aim

This study aimed to examine gender and study progression differences in medical students’ mental health and perceived study demands. Specifically, we addressed the following questions:

1. Do men and women differ in their reported mental health and perceived study demands?
2. Do beginners and advanced students differ in their reported mental health and perceived study demands?

## Methods

### Study design and setting

We conducted a cross-sectional survey at the authors’ affiliations – medical faculties of two German universities – across three data collection waves in the winter terms of the academic years 2021, 2022, and 2023. The Charité – Universitätsmedizin Berlin (Charité) is in a major German city (population >1 million) and organises its medical curriculum according to a reformed model that emphasises early patient contact and communicative and methodological competencies. The Friedrich-Alexander Universität Erlangen-Nürnberg (FAU) is in a medium-sized German city (population >100,000) and follows the traditional medical curriculum, which separates the preclinical phase, focusing on biomedical and anatomical basics in years 1–2, from the clinical phase, with applied subjects and patient contact in years 3–5. Both curricula conclude with a practical year six, completed in three four-month rotations, followed by written and oral state examinations. Specialist medical training usually follows on from the taught university phase. Both universities offered a mix of online courses and in-person teaching between summer term 2020 and winter term 2021 due to COVID-19 pandemic-related restrictions, but returned to full in-person teaching by summer term 2022.

### Measures and definitions

Depression is “a period of at least [two] weeks during which there is either depressed mood or the loss of interest or pleasure in nearly all activities” [34, S. 349]. We used the two-item German version of the Patient Health Questionnaire (PHQ-2) [35] to measure depression with its two leading symptoms, loss of interest and depressed mood. The PHQ-2 has comparable psychometric properties to the longer versions, e.g. Cronbach’s α = .73 and convergent validity (*r* = .53) with the Beck’s Depression Inventory [36], but is more economical to answer.

Burnout is a work-related phenomenon defined as “an exhaustion due to prolonged exposure to work-related problems” [37, S. 95]. We measured the dimension exhaustion defined as “a consequence of intensive physical, affective, and cognitive strain, i. e., as a longterm consequence of prolonged exposure to certain job demands” [38, S. 14] using the student version of the Oldenburg Burnout Inventory (OLBI-S) with high values indicating high exhaustion. The OLBI-S has satisfying psychometric properties, e.g. Cronbach’s α = .87 [39], and was developed in German. The focus on exhaustion as the core variable of burnout allows a concise assessment of study-related strain and early signs of burnout among medical students, who are particularly exposed to high workload and emotional demands. Numerous studies have demonstrated that this subscale alone provides a reliable and valid indicator of burnout risk and psychological strain [40,41]. Therefore, for economic and content-related reasons, it is common to use only this dimension [42] or interpret the subscales separately [43].

The prevalence of psychiatric diagnoses describes the presence of psychological impairments. Based on the German Work Ability Index (WAI) [44] we asked participants to identify illnesses or injuries and to indicate whether it has been medically diagnosed or treated with the option “psychological impairments (e.g. depression, anxiety, chronic insomnia, psycho-vegetative fatigue syndrome)” to measure the prevalence using the coding *no diagnosis* (1), *self-diagnosed* (2), or *diagnosed by a professional* (3).

Study demands describe elements of the institutional learning environment that interact with students’ abilities and characteristics to influence success or dropout rates. We used the German Messinstrument für die Wahrnehmung von Studienbelastungen (“Measuring Instrument for the Perception of Study Demands”, MWS) [45] to measure perceived study demands in six sub-dimensions: mode of science, learning activities, self-structuring, contact and cooperation, performance pressure and failure, and combining theory and practice. Internal consistencies for these dimensions range from .69 to .80 [45].

Study satisfaction refers to the students’ appraisal of their studies. We used the self-devised single item “how satisfied are you overall with your study?” measured on a five-point Likert scale ranging between -- (1) and ++ (5) [46].

Satisfaction with learning progression describes students’ reflective perception of and satisfaction with their academic growth and achievements over time, considering their own input and the resulting learning outcomes. We used the self-devised single item “how satisfied are you with the learning progress you have achieved so far this term?” measured on a five-point Likert scale ranging between *not at all satisfied* (1) and *completely satisfied* (5).

### Procedure

**Survey administration**. We collected data in three waves during the winter terms of the academic years 2021, 2022, and 2023 using an online survey and convenience sampling. The survey was administered separately at both universities on their respective in-house servers in accordance with European data protection laws. Students were invited via mailing lists from the respective deans of studies and, with assistance from the student unions, utilising their social media presence. About four weeks after the initial invitation, we sent a reminder for participation across recruitment channels. This procedure ensured pseudonymous participation without complex bureaucratic processes, which were unfeasible because of resource constraints. However, we cannot confidently assess how many students we reached with these recruitment efforts.

**Inclusion and exclusion criteria**. We included all medical students at Charité or FAU. Exclusion criteria were study subjects other than medicine, incomplete datasets with fewer than 20% answered questions, less complete datasets in cases of multiple participations within one data collection period, and missing gender values. Each participant was included with only one dataset across the three data collection waves; i.e., for students participating at multiple waves, we selected the most complete dataset and excluded all other participations to ensure independence of data across the data collection waves.

**Ethical considerations**. We obtained the ethics committee’s assessment that no detailed examination of our study was necessary because of the pseudonymous nature of our design (vote 21-393-ANF from 21 November 2021). We carried out the study in accordance with the Declaration of Helsinki, which includes the guarantee of anonymity of participants. We obtained informed consent for all participants. Since our data collection was exploratory in nature, we did not perform a sample size calculation.

**Statistical analysis**. We analysed data using Microsoft Excel Version 2019 and IBM SPSS Version 29. We transformed the scores for the constructs depression, emotional exhaustion, and perceived study demands into percentage of maximum possible (POMP) scores using the formula

\[
\left(\frac {(x_{i}-{\rm min})} {({\rm max}-{\rm min})}\right) * 10
\]

where *x_i_* are individual observed values, *max* is the maximum scale value, and *min* is the minimum scale value [47]. The resulting POMP scores range from 0 to 10, which makes them intuitively interpretable compared to the scales’ original scores (e.g., 0 to 3 for the PHQ-2 [35] or 1 to 5 MWS [45]). Simultaneously, the linear transformation does not affect the scales’ properties, i.e. the same parametric analyses can be performed without constraints [47]. To answer our research questions, we used descriptive statistics, *t*-tests for comparisons between two groups, χ^2^-tests for differences on nominal variables, and ANOVAs. We used Bonferroni-Holm corrections to adjust for multiple comparisons, with p-values indicating statistical significance. Pairwise deletion was implemented for missing data, resulting in larger, but varying, sample sizes for each analysis compared to listwise deletion [48]. Later items in the survey had a higher likelihood of missing values because participants terminated the survey prematurely. However, this procedure did not introduce bias to our data.

## Results

### Participant characteristics

At Charité, 1,755 visitors to the survey start page were recorded across all three waves. We immediately excluded 635 visitors who did not consent to participate or who provided a study subject other than medicine. Two hundred eight datasets were excluded because the respondents provided answers to <20% of the questions. Sixty-one datasets of students who participated multiple times within one data collection wave were excluded. We excluded 16 students who did not identify their gender. Lastly, 155 student datasets were excluded because they participated in another wave. After data exclusion, 680 medical students remained in the dataset for Charité, n = 300, n = 180, and n = 200 by survey wave. With approximately 4,800 medical students enrolled at Charité, the response rates were 6%, 4%, and 4% across the three survey waves, assuming all students saw our invitation to participate.

**Figure 1:**
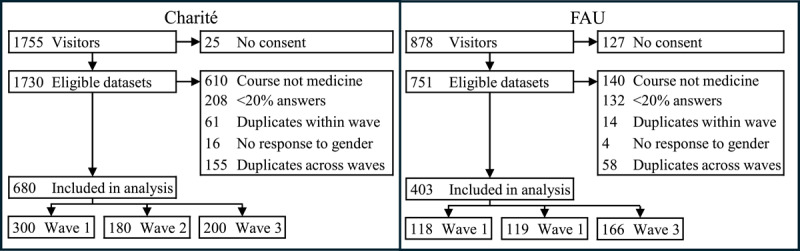
Study flowchart.

At FAU, 878 visitors to the survey start page were recorded across all three waves. We immediately excluded 267 visitors who did not consent to participate or who provided a study subject other than medicine. One hundred thirty-two datasets were excluded, because the respondent provided answers to <20% of questions. Fourteen datasets of students who participated multiple times within one data collection wave were excluded. We excluded four students who did not identify their gender. Lastly, 58 student datasets were excluded because they participated in another wave. After data exclusion, 403 medical students remained in the dataset for FAU, n = 118, n = 119, and n = 166 by survey wave. With approximately 2,800 medical students enrolled at FAU, the response rates were 4%, 4%, and 6% across the three survey waves, assuming all students saw our invitation to participate. Figure 1 displays a participant flow diagram for both universities, and Table 1 shows sample characteristics for each survey wave by university. For the following analyses, we pooled all data into one dataset.

**Table 1:** Sample characteristics.


VARIABLE	CHARITÉ			FAU		
	
	t_1_	t_2_	t_3_	t_1_	t_2_	t_3_

N	300	180	200	118	119	166

Gender (N, % female)	216 (72%)	120 (67%)	143 (72%)	95 (81%)	89 (75%)	128 (77%)

Age (M, SD)	25.03 (5.24)	24.73 (4.36)	24.49 (4.01)	23.45 (3.47)	22.82 (3.81)	23.30 (3.90)

Term (M, SD)	5.39 (3.18)	5.54 (3.21)	5.38 (3.41)	5.07 (2.67)	3.03 (1.25)	3.80 (2.55)


*Note*. FAU = Friedrich-Alexander Universität Erlangen-Nürnberg; t_1_ = winter term 2021/22; t_2_ = winter term 2022/23; t_3_ = winter term 2023/24.

### Mental health and perceived study demands

Our first research question aimed to assess gender differences in mental health, study demands, and satisfaction with the study and learning. Table 2 presents descriptive statistics and t-tests comparing men and women, pooled across survey waves and both universities. Men reported lower mental health and perceived study demands than women across five of six dimensions. Regarding exhaustion as a dimension of burnout, 376/886 respondents (42%) reported high-risk scores (≥23 on the OLBI), with fewer men (29%) than women (47%) reporting values above the threshold. Regarding depression, 274/1031 respondents (27%) reported depressive symptoms above the clinically relevant threshold (≥3 for the PHQ-2 [36]), with fewer men (21%) reporting values above the threshold than women (29%). Despite differences in mental health and study demands, no gender effect was found in satisfaction with their studies or learning.

**Table 2:** Gender differences regarding depression, emotional exhaustion, six dimensions of study demands, study satisfaction, and satisfaction with learning.


	α	MEN			WOMEN			*t*	*p*	*d*
	
		N	M	SD	N	M	SD			

Mental health										

Depression	.83	249	2.90	2.66	697	3.49	2.76	**–2.91**	**.004**	**–0.22**

Exhaustion	.91	252	4.77	2.13	705	5.64	2.07	**–5.72**	**.000**	**–0.42**

Study demands										

Mode of Science	.89	253	3.73	2.25	700	4.54	2.36	**–4.71**	**.000**	**–0.35**

Combining theory and practice	.84	255	3.45	2.01	706	4.02	2.18	**–3.63**	**.000**	**–0.26**

Learning activities	.89	256	4.78	2.20	708	5.53	2.22	**–4.68**	**.000**	**–0.34**

Performance pressure and failure	.82	253	3.83	2.22	707	4.98	2.20	**–7.14**	**.000**	**–0.52**

Contact and cooperation	.86	256	4.14	2.21	708	4.50	2.33	–2.18	.030	–0.16

Self-structuring	.86	256	4.77	2.54	707	5.35	2.39	**–3.30**	**.001**	**–0.24**

Satisfaction										

Study satisfaction	–	268	7.15	2.53	741	6.75	2.25	**2.40**	**.017**	**0.17**

Satisfaction with learning	–	263	6.18	2.81	733	5.82	2.72	1.83	.068	0.13


*Note*. α = Cronbach’s alpha; statistically significant results are displayed in bold following Bonferroni-Holm adjustments for ten *t*-tests.

Consistent with the t-test results, we found gender effects on mental health and most study demands, but not on satisfaction with study or learning, in 2x2 ANOVAs with gender and university as factors (Supplementary Table S1). We observed differences between the two universities in five study demands favouring Charité. We did not find statistically significant interaction effects between gender and university for any outcome.

Table 3 shows results for (self-)diagnosed psychological disorders stratified by gender and university. Women report professional diagnoses with higher relative frequency than men in both universities (25% and 22% vs. 17% and 8%). Relative frequencies for self-diagnoses are comparable for gender and university. Men report no diagnoses more frequently than women at both universities (68% and 81% vs. 59% and 63%, respectively).

**Table 3:** Crosstables and χ^2^-tests for gender differences regarding (self-)diagnoses with psychological disorders for the two universities.


	TOTAL		MEN		WOMEN		*χ* ^2^	*p*	*v*
		
	N	%	N	%	N	%			

**Charité**							4.31	.116	.09

Not diagnosed	342	60%	110	66%	232	58%			

Self-diagnosed	100	18%	28	17%	72	18%			

Professionally diagnosed	125	20%	28	17%	97	24%			

**FAU**							**6.89**	**.032**	**.14**

Not diagnosed	242	71%	63	81%	179	68%			

Self-diagnosed	55	16%	11	14%	44	17%			

Professionally diagnosed	46	13%	4	5%	42	16%			


*Note*. FAU = Friedrich-Alexander Universität Erlangen-Nürnberg.

Our second research question addressed differences between beginners and advanced students regarding their mental health, study demands, and satisfaction with their study and learning. Table 4 presents descriptive statistics and t-tests for the two cohorts, pooled across survey waves and both universities. We did not find differences regarding mental health for progression. However, advanced students reported lower study demands in dealing with performance pressure and self-structuring as well as lower study satisfaction than beginners. We did not find statistically significant differences on the other scales.

**Table 4:** Differences between beginners and advanced students regarding depression, emotional exhaustion, six dimensions of study demands, study satisfaction, and satisfaction with learning.


	BEGINNERS			ADVANCED			*t*	*p*	*d*
	
	N	M	SD	N	M	SD			

Mental health									

Depression	387	3.37	2.61	198	2.93	2.65	1.91	.056	0.17

Exhaustion	395	5.19	2.15	199	5.43	1.94	–1.35	.178	–0.12

Study demands									

Mode of Science	384	4.35	2.29	204	4.08	2.31	1.35	.179	0.12

Combining theory and practice	390	3.80	2.08	205	3.67	2.20	0.71	.479	0.06

Learning activities	392	5.45	2.20	205	4.96	2.12	2.64	.009	0.23

Performance pressure and failure	390	4.97	2.26	205	4.07	2.26	**4.60**	**.000**	**0.40**

Contact and cooperation	392	4.43	2.39	205	4.16	2.23	1.31	.192	0.11

Self-structuring	391	5.42	2.54	205	4.47	2.04	**4.63**	**.000**	**0.40**

Satisfaction									

Study satisfaction	417	7.34	2.10	209	6.41	2.48	**4.89**	**.000**	**0.41**

Satisfaction with learning	412	6.09	2.79	206	5.89	2.51	0.90	.371	0.08


*Note*. Statistically significant results are displayed in bold following Bonferroni-Holm adjustments for ten *t*-tests.

Results of the 2x2 ANOVA with university and study progression as factors did not produce a robust pattern (Supplementary Table S2). Beginners reported greater difficulty with performance pressure and self-structuring, two dimensions of perceived study demands, but also higher study satisfaction than advanced students. A main effect for university was found only for the combination of theory and practice as a single dimension of study, favouring Charité. The interaction term between university and progression showed differences for emotional exhaustion, contact and cooperation, and self-structuring as two dimensions of study demands.

## Discussion

This study aimed to investigate the effects of gender and study progression on mental health and study demands in medical students at two German universities. We found consistent evidence for gender effects, with women reporting poorer outcomes regarding mental health and perceived study demands than men. Most strikingly, burnout prevalence was twice as high in women as in men. Additionally, women reported a higher prevalence of professional diagnosis with a psychological disorder. Indeed, meta-analyses have found the prevalence of mood disorders among women to be 1.5 to 2 times higher than for men [49] with higher odds ratios in adolescence and in countries with greater gender equity [50]. Recent studies with students report similar tendencies [51]. Thus, our study supports the body of literature that reports a higher prevalence of affective mental health difficulties in women, with particular focus on depression and emotional exhaustion as a dimension of burnout in students [11]. Our results are thus consistent with the discourse on gender roles, particularly the view that they disadvantage women and/or protect men [17]. However, we cannot assess the exact impact of gender roles on our sample, as we did not survey conceptions of femininity and masculinity.

Men reported lower perceived study demands in five of six dimensions, e.g., performance pressure and failure or learning activities. Other studies did not find such a consistent gender effect using the same scale [45], but instead reported slight gender differences favouring men [51] or no gender differences [52,53]. However, these studies addressed study demands across different faculties without a focus on medical students or other countries, or without an explicit focus on gender differences. High correlations between difficulties with study demands and exhaustion and depression suggest that these constructs are closely related [46]. Thus, it is not surprising that women with higher odds ratios of experiencing poor mental health also report difficulties in coping with study demands. Supporting students’ mental health by offering interventions such as counselling, mentoring programmes, or peer groups [54,55] is thus also likely to improve students’ ability to cope with study demands, thereby entering a virtuous cycle of support. A combination of behavioural, individual-centred approaches and situational, system-centred approaches is most likely to achieve a resilient student body [54].

We did not find differences in coping with study demands between beginners (terms 1 to 3) and advanced students (terms 8 to 10). These findings contrast with earlier studies reporting that beginners have greater difficulties with learning activities or exam performance [46] or with perceived stress [56]. Indeed, entering university is a time of change on multiple levels, with many students moving out and to other cities, leaving friends and family for new social contacts and being introduced to the codes of academia [57]. Thus, beginners should perceive more stress than their more experienced counterparts. However, the stress could be limited to the first term, and students can quickly become familiar with university, learning, and fitting in [56]. Merging students from the first three terms into the group of beginners might have diluted the negative effects of the early adoption phase in the first term. This problem also arises when surveying first-year students – as others have operationalised the entry phase [9,52] – who are diverse in their experience depending on when the survey is conducted [58].

We did not find effects of study progression regarding mental health. This null finding is in line with longitudinal studies that showed little to no change over time in mental health [29,59]. However, both meta-analyses on depression [10] and burnout [11] cautiously reported declining trends of mental health difficulties with study progression. In contrast, Rotenstein et al. [33] found advanced students struggling *more* with depression and exhaustion than beginners.

### Strength and limitations

This study benefits from multi-year data collection across three waves, enabling a more comprehensive assessment of trends in medical students’ mental health and study demands. Additionally, we examined a broad range of study-related stressors, providing a nuanced picture of academic strain. Despite these strengths, our study relies on convenience samples from two universities, with participants recruited via student union mailing lists and social media. While this may introduce selection bias – potentially attracting students with stronger opinions on study-related stress – the gender ratio, age distribution, and median semester closely reflect the overall student populations at both institutions. We are therefore confident that our findings offer meaningful insights. However, differences between universities extend beyond their degree programs to geographic, institutional, and socio-economic contexts, which may have influenced the results. The use of self-reported data and some self-developed items (e.g., for study satisfaction) also carries risks of subjective bias and response tendencies. Our design, as a cross-sectional survey, did not control for confounders as randomised controlled trials would have. Finally, the long-term impact of the COVID-19 pandemic and varying institutional responses may have shaped students’ experiences, potentially affecting our findings. Future research should account for these contextual factors and consider longitudinal designs to better capture changes over time.

## Conclusions

Our findings highlight persistent gender differences in mental health and perceived study demands among medical students, with women consistently reporting higher levels of mental health and perceived study demands. These results underscore the need for targeted interventions —i.e., identifying the groups most in need —to support medical students’ mental well-being, with a particular focus on gender-sensitive measures and the challenges faced at different stages of medical education. Participatory, student-led peer-to-peer approaches reduce barriers and can be combined with on-campus professional counselling. Future research should explore underlying mechanisms, such as coping strategies, diverse conceptions of masculinity and femininity, as well as institutional factors such as curriculum density, student-teacher ratios, spatial layouts regarding commuting and retreat areas, or the existence of dedicated liaison lecturers and student health management departments, to develop more effective support systems.

## Additional File

The additional file for this article can be found as follows:

10.5334/pme.1899.s1 Supplementary File 1. Supplementary Tables S1–S2.
