# Supplementary material for: Gender Disparities in Mental Health and Study Demands among Medical Students: A Three-Cohort Study at Two German Universities
Source: Perspect Med Educ. 2025 Dec 4;14(1):927–38. doi: 10.5334/pme.1899 (PMC12686339; doi:10.5334/pme.1899)
Supplement: Supplementary File 1. — Supplementary Tables S1–S2. [file pme-14-1-1899-s1.pdf]

Table S1

Gender differences regarding depression, emotional exhaustion, six dimensions of study demands, study satisfaction, and satisfaction with learning for the two universities

Constructs	Independent Variables	Sum of squares	*F*	*p*	η^2	R^2
Depression	Uni	18.12	2.43	.119		.02
	Gender	59.98	8.04	.005	.008	
	Uni*gender	4.28	0.57	.449		
Exhaustion	Uni	19.82	4.58	.033		.04
	Gender	162.30	37.51	.000	.038	
	Uni*gender	15.80	3.65	.056		
Mode of Science	Uni	43.33	8.05	.005	.008	.03
	Gender	89.58	16.64	.000	.017	
	Uni*gender	1.77	0.33	.566		
Combining theory and practice	Uni	204.72	48.16	.000	.048	.08
	Gender	43.87	1.32	.001	.011	
	Uni*gender	2.92	0.69	.407		
Learning activities	Uni	48.30	9.93	.002	.010	.03
	Gender	118.03	24.27	.000	.025	
	Uni*gender	3.29	0.68	.411		
Performance pressure and failure	Uni	24.23	5.01	.025		.06
	Gender	198.71	41.08	.000	.041	
	Uni*gender	1.29	0.27	.606		
Contact and cooperation	Uni	107.00	2.69	.000	.021	.03
	Gender	1.99	2.13	.145		
	Uni*gender	4.27	0.83	.364		
Self-structuring	Uni	298.89	53.83	.000	.053	.07
	Gender	32.39	5.83	.016		
	Uni*gender	3.49	0.63	.428		
Study satisfaction	Uni	15.14	2.81	.094		.01
	Gender	21.75	4.03	.045		
	Uni*gender	.70	0.13	.720		
Satisfaction with learning	Uni	27.93	3.76	.053		.02
	Gender	15.20	2.04	.153		
	Uni*gender	17.72	2.38	.123		

Note. df = 1 for all ANOVAs; values for eta are only displayed if corresponding $p \leq .005$

Table S2

Differences between beginners and advanced students regarding depression, emotional exhaustion, six dimensions of study demands, study satisfaction, and satisfaction with learning for the two universities

Constructs	Independent Variables	Sum of squares	F	p	η²	R²
Depression	Uni	0.27	0.04	.844		.01
	Progression	13.21	1.92	.167		
	Uni*progression	6.29	0.91	.340		
Exhaustion	Uni	0.06	0.01	.903		.03
	Progression	22.00	5.21	.023		
	Uni*progression	51.12	12.12	.001	.020	
Mode of Science	Uni	27.74	5.29	.022		.01
	Progression	1.26	0.24	.625		
	Uni*progression	0.15	0.03	.864		
Combining theory and practice	Uni	114.06	26.74	.000	.043	.05
	Progression	3.38	0.79	.374		
	Uni*progression	0.80	0.19	.666		
Learning activities	Uni	17.75	3.87	.050		.05
	Progression	17.65	3.85	.050		
	Uni*progression	29.93	6.53	.011		
Performance pressure and failure	Uni	5.43	1.06	.303		.04
	Progression	53.32	10.43	.001	.017	
	Uni*progression	8.29	1.62	.203		
Contact and cooperation	Uni	14.60	2.76	.097		.04
	Progression	14.24	2.69	.102		
	Uni*progression	42.93	8.10	.005	.013	
Self-structuring	Uni	32.77	6.15	.013		.09
	Progression	114.61	21.52	.000	.035	
	Uni*progression	71.71	13.47	.000	.022	
Study satisfaction	Uni	28.72	5.82	.016		.05
	Progression	163.61	33.12	.000	.051	
	Uni*progression	37.55	7.60	.006		
Satisfaction with learning	Uni	14.49	2.01	.157		.02
	Progression	6.39	0.89	.347		
	Uni*progression	17.71	2.46	.117		

Note. df = 1 for all ANOVAs; values for eta are only displayed if corresponding p ≤ .005
